# COVID-19 patients with negative results on initial screening: Experience of Brunei Darussalam

**DOI:** 10.5365/wpsar.2022.13.1.772

**Published:** 2022-01-06

**Authors:** Vui Heng Chong, Justin Wong, Muhammad Syafiq Abdullah, Rosmonaliza Asli, Riamiza Natalie Momin, Siti Nabilah Ahmed, Norhasyimah Tamin, Babu Ivan Mani, Pui Lin Chong

**Affiliations:** a Department of Medicine, Raja Isteri Pengiran Anak Saleha Hospital, Bandar Seri Begawan, Brunei Darussalam.; b Department of Medicine, Pengiran Muda Mahkota Pengiran Muda Haji Al-Muhtadee Billah Hospital, Tutong, Brunei Darussalam.; c Pengiran Anak Puteri Rashidah Sa’adatul Bolkiah Institute of Health Sciences, Universiti Brunei Darussalam, Bandar Seri Begawan, Brunei Darussalam.; d Department of Public Health, Ministry of Health, Bandar Seri Begawan, Brunei Darussalam.

## Abstract

In any infectious disease outbreak, early diagnosis, isolation of cases and quarantine of contacts are central to disease containment. In Brunei Darussalam, suspected cases of coronavirus disease 2019 (COVID-19) were quarantined either at home or at designated centres and were tested immediately for severe acute respiratory syndrome coronavirus 2. We report on 10 cases of COVID-19 that initially tested negative for COVID-19 and were positive on re-testing after becoming symptomatic. These cases comprised 3.8% of the 266 total confirmed COVID-19 cases in Brunei Darussalam as of 9 July 2021, when this study was conducted. All the cases were in quarantine at home and were tested early during their quarantine period. Since then, home quarantine has been replaced by quarantine at designated centres only, with testing on the 12th day of quarantine.

The pandemic of coronavirus disease 2019 (COVID-19) caused by severe acute respiratory syndrome coronavirus 2 (SARS-CoV-2) continues, and, as of 2 November 2021, the cumulative number of cases reported globally was over 246 million and the cumulative number of deaths nearly 5 million. (*1*) In any infectious disease outbreak, early diagnosis, contact tracing and effective quarantine and isolation are central to disease containment. (*2*-*4*) The reverse transcription polymerase chain reaction (RT–PCR) assay is currently the most reliable test for diagnosis of COVID-19 during the symptomatic phase; (*4*) however, testing a patient too early after infection can lead to false-negative results. (*5*, *6*)

On 9 July 2021, when this study was conducted, there were 266 confirmed COVID-19 cases in Brunei Darussalam: 8 patients remained in the National Isolation Centre, 255 patients recovered and 3 died. (*7*) According to the World Health Organization (WHO), Brunei Darussalam remained at national transmission assessment stage 1, with only imported cases, from 6 May 2020 to 7 August 2021, when locally transmitted cases again began to be reported. (*7*, *8*) Suspected cases were defined according to the WHO definition, (*9*) and incoming travellers were placed in quarantine, either at home or at designated quarantine centres. Testing was conducted only with RT–PCR immediately upon commencement of quarantine.

We report 10 confirmed COVID-19 cases that initially tested negative in quarantine and the impact of this finding on our protocols during the first month of the COVID-19 outbreak in Brunei Darussalam.

## Methods

A descriptive study was conducted of data retrieved from a prospectively maintained Excel® database that was created to monitor COVID-19 patients. COVID-19 cases (*n* = 12) with initial negative tests within 14 days (the maximum incubation period of SARS-CoV-2) of diagnosis of COVID-19 were included. Two patients were excluded, as the initial negative tests were performed > 14 days (24 and 36 days) before the day of diagnosis.

The data extracted for analyses were age, sex, number and dates of RT–PCR tests before admission, symptoms, disease category, outcomes and possible source of infection.

### Ethics statement

This study was conducted in accordance with the provisions of the Declaration of Helsinki.

## Results

### Cases

Ten (3.8%) suspected cases (median age, 41.5 years; range, 28–72; 4 males and 6 females) had a negative COVID-19 test before testing positive (**Table 1**), with a median of 5 days (range, 1–10) between the negative and positive tests (**Table 1**). All 10 cases were initially tested because they were contacts of confirmed COVID-19 cases, and all were quarantining at home.

**Table 1:** Characteristics and outcomes of cases with initial negative tests before testing positive for COVID-19

Case (cluster)	Age/sex	Number of initial negative tests for SARS-CoV-2	Number of days between index positive confirmation and testing	Number of days between negative test and positive test	Indication for repeat testing (days after initial test)	Disease severity	Possible source of infection
1 (A)	45/F	1	1	3	Symptoms (3)	Mild	All cases were contacts of the index case (spouse of case 5) who had returned from an overseas trip
2 (A)	33/M	1	1	4	Symptoms (3)	Mild	
3 (A)	50/F	1	1	4	Symptoms (4)	Moderate	
4 (A)	51/F	1	1	4	Symptoms (4)	Mild	
5 (A)	72/M	1	0	6	Asymptomatic, family request	Mild	
6 (A)	43/M	1	0	6	Symptoms (5)	Mild	
7 (A)	36/F	2	1	7 10	Spouse (case 6) tested positive Symptoms (7)	Mild	
8 (B)	40/F	1	0	7	Symptoms (7)	Mild	Contact of two confirmed cases: spouse (admitted 7 days earlier) and daughter (admitted 5 days earlier)
9 (C)	28/M	1	0	8	Symptoms (4)	Mild	Sibling was a confirmed case
10 (D)	39/F	1	2	1	Persistent symptoms (1)	Mild	Contact of two confirmed cases: spouse (admitted 3 days earlier) and son (admitted 2 days earlier)

Cases 1–7 were in a family cluster (cluster A) of 15 confirmed COVID-19 cases. Three cases within this cluster had returned from vacations on three separate flights, two flights arriving 5 and 16 days after the first flight. The index case in this cluster was the spouse of case 5, who returned home on the first flight and developed symptoms soon after arrival. She had been symptomatic for 3 days before being tested and was confirmed positive for SARS-CoV-2. All the subsequent cases that tested negative before testing positive, except case 5, developed symptoms during home quarantine after an initial negative test. Case 5 was retested at the request of the family, and, although remaining asymptomatic, also retested positive. Case 7 (spouse of case 6) had two negative tests: initially as a contact of the index case and then when case 6 retested positive. She tested positive on the third test, conducted after the onset of symptoms.

Case 8 (cluster B, 25 cases) was a contact of her spouse and daughter, who had tested positive for SARS-CoV-2 earlier. She later developed symptoms and retested positive 7 days after her initial negative test. Her spouse had contracted the infection from a friend.

Case 9 (cluster C, 7 cases) was a contact of a sibling who had returned from travel. The second test was conducted because of persistent symptoms.

Case 10 (cluster D, 14 cases) was a contact of her spouse, who contracted the infection from a work colleague. Two days after an initial negative test, case 10 was retested because of persistent symptoms and tested positive.

All the cases except case 3 were categorized as mild. Two cases (cases 8 and 9) were hospitalized twice when they retested positive after discharge.

### Impact on protocols

Two notable changes were made to the COVID-19 protocols in Brunei Darussalam during this period. First, home quarantine was abolished for incoming travellers within 1 month of the outbreak on 20 March 2020. Second, the testing schedule during quarantine was revised for local suspected cases from immediate testing only to testing on the 4th day of quarantine.

## Discussion

In our study, 3.8% of COVID-19 cases in Brunei Darussalam as of 9 July 2021 initially tested negative. Initial negatives or delayed positives are a concern if they are missed cases that become vehicles for continued COVID-19 spread. (*10*) Fortunately, these 10 cases were under the mandatory 14-day quarantine and had repeat tests, usually because of development of symptoms. No additional transmission was linked to these 10 cases.

Our experience confirms that testing early, especially during the pre-symptomatic phase, can lead to false-negative results. (*11*, *12*) Therefore, our testing protocol was revised several times, from immediate testing of cases with initial negative results to the current (as of December 2020) testing on the 12th day of quarantine. Cases are still tested if they become symptomatic or if symptoms persist. Incoming travellers are tested according to a schedule based on the countries to which they had travelled (travel passes A, B and C). Testing can be done immediately or 1 day after arrival from countries listed for travel pass A under certain conditions (pre-travel approved entry permit, COVID-19 testing within 72 hours of arrival, payment for post-arrival RT–PCR and a health tracking application installed on mobile phone), on the 5th day of quarantine for travel pass B and on the 12th day of quarantine for travel pass C.

Home quarantine for returned travellers presented some difficulty in monitoring without personnel on site or a remote monitoring system. All patients were given an information leaflet and a designated number to call if required, for example if they developed symptoms. It is possible that some patients may not call the number, because of unawareness, having misplaced the information leaflet or choosing not to inform the authorities of the progress of their illness. There were also logistical issues for those who required retesting, because of lack of personnel for testing in home quarantine. Quarantine in designated centres was found to be preferable, as COVID-19 is easier to monitor in known locations, of which there are only a few. In home quarantine, quarantine regulations may not be adhered to strictly, and continued transmission can occur.

Our findings show that the pandemic situation is fluid and that protocols and processes must therefore be revised continually. This is particularly important as the pandemic is complicated by SARS-CoV-2 variants of concern. Missed cases are the driver of continued disease transmission. At the time this study was conducted, Brunei Darussalam had remained at WHO stage 1 of transmission (imported cases only) for over one year, (*7*, *8*) and we have continued to maintain all preventive and monitoring measures while simultaneously adjusting our protocols and processes. Our findings can be generalized to other settings, especially those in which containment is still possible.

In conclusion, our experience highlights the importance of monitoring people in quarantine and revising protocols to control outbreak situations. Undetected and missed cases can lead to continued disease transmission. Since home quarantine was abolished and testing of those in quarantine was delayed to later in the quarantine period, we have not registered any further COVID-19 cases with initial negative tests. The lessons learnt can be applied in other countries and to outbreaks of other infectious diseases.
